# Bactericidal Action of Photogenerated Singlet Oxygen from Photosensitizers Used in Plaque Disclosing Agents

**DOI:** 10.1371/journal.pone.0037871

**Published:** 2012-05-22

**Authors:** Kirika Ishiyama, Keisuke Nakamura, Hiroyo Ikai, Taro Kanno, Masahiro Kohno, Keiichi Sasaki, Yoshimi Niwano

**Affiliations:** 1 Tohoku University Graduate School of Dentistry, Sendai, Japan; 2 Graduate School of Bioscience and Biotechnology, Tokyo Institute of Technology, Tokyo, Japan; Glasgow University, United Kingdom

## Abstract

**Background:**

Photodynamic therapy (PDT) has been suggested as an efficient clinical approach for the treatment of dental plaque in the field of dental care. In PDT, once the photosensitizer is irradiated with light of a specific wavelength, it transfers the excitation energy to molecular oxygen, which gives rise to singlet oxygen.

**Methodology/Principal Findings:**

Since plaque disclosing agents usually contain photosensitizers such as rose bengal, erythrosine, and phloxine, they could be used for PTD upon photoactivation. The aim of the present study is to compare the ability of these three photosensitizers to produce singlet oxygen in relation to their bactericidal activity. The generation rates of singlet oxygen determined by applying an electron spin resonance technique were in the order phloxine > erythrosine ≒ rose bengal. On the other hand, rose bengal showed the highest bactericidal activity against *Streptococcus mutans*, a major causative pathogen of caries, followed by erythrosine and phloxine, both of which showed activity similar to each other. One of the reasons for the discrepancy between the singlet oxygen generating ability and bactericidal activity was the incorporation efficiency of the photosensitizers into the bacterial cells. The incorporation rate of rose bengal was the highest among the three photosensitizers examined in the present study, likely leading to the highest bactericidal activity. Meanwhile, the addition of L-histidine, a singlet oxygen quencher, cancelled the bactericidal activity of any of the three photoactivated photosensitizers, proving that singlet oxygen was responsible for the bactericidal action.

**Conclusions:**

It is strongly suggested that rose bengal is a suitable photosensitizer for the plaque disclosing agents as compared to the other two photosensitizers, phloxine and erythrosine, when used for PDT.

## Introduction

Photodynamic therapy (PDT) has been suggested as an efficient clinical approach for the treatment of different types of cancer [1]–[3] and as an alternative to chemical disinfection agents [4]–[7]. In the field of dental care, PDT has been applied to treat endodontic infection including dental plaque [8]–[15], which is structurally and functionally organized multi-species biofilm colonizing tooth surfaces and epithelial cells covering gingival sulcus and periodontal pocket [16].

The PDT consist of mainly there components. These are light, oxygen, and a photosensitizer. Once the photosensitizer is irradiated with light of a specific wavelength, it absorbs photons of the wavelength and transfers the excitation energy to molecular oxygen which is in turn metamorphosed to its diamagnetic form, singlet oxygen [17] with other reactive oxygen species (ROS) formed downstream such as superoxide anion, hydroxyl radicals, and hydrogen peroxide [18]–[20]. Of the ROS, singlet oxygen plays a central role for cytotoxicity in PDT [21], [22], indicating that the larger amount of singlet oxygen the target is exposed to, the more effectively undesired cells such as cancer cells and bacteria are killed.

Plaque disclosing agents, which are used to detect plaques on the tooth surface, usually contain photosensitizers such as rose bengal, erythrosine, and phloxine, all of which are also used for food coloring. Thus, if these plaque disclosing agents are irradiated with light of a suitable wavelength for the photosensitizers, singlet oxygen is validly and locally generated around the plaques disclosed by the agents. The aim of the present study is to compare the ability of these three photosensitizers to produce singlet oxygen upon irradiation with the light at around their maximal absorption wavelengths in relation to their bactericidal activity. To determine the photogenerated singlet oxygen, an electron spin resonance (ESR) technique was applied based on previous studies showing that the relative yield of singlet oxygen generated by various photosensitizers can be evaluated properly by ESR analysis [23]–[25]. To clarify the relation between the singlet oxygen producing ability and the bactericidal activity, incorporation efficiency of these photosensitizers into bacterial cells was also examined.

## Results

### Determination of photogenerated singlet oxygen by ESR analysis of nitroxide radical

**Figure 1:**
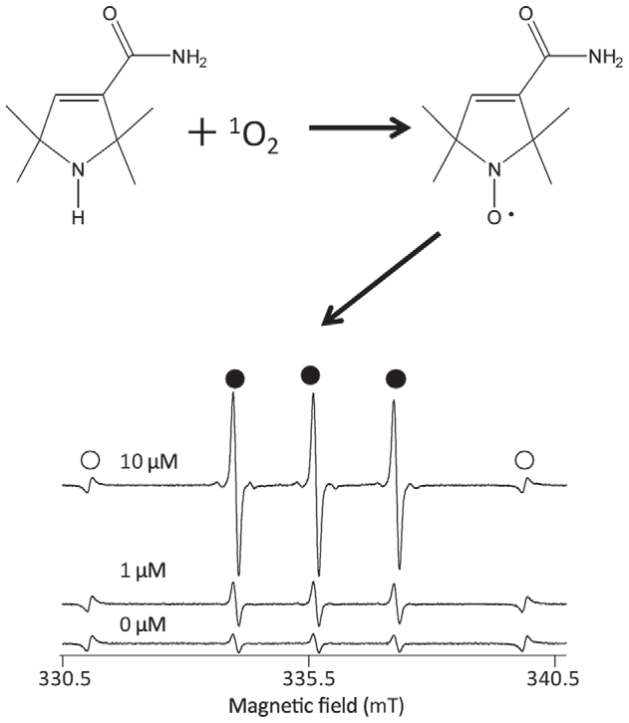
A schema showing oxidation of a sterically hindered amine TPC (2,2,5,5-tetramethyl-3-pyrroline-3-carboxamide) by singlet oxygen to give rise to the nitroxide radical, and representative ESR spectra of the laser-irradiated mixtures containing rose bengal and a sterically hindered amine TPC. The mixture containing 450 mM TPC and 0, 1 or 10 µM rose bengal was irradiated by the laser light with a wavelength of 532 nm for 60 s. Open circle and closed circle indicate Mn^2+^ marker and the nitroxide radical, respectively.

**Figure 2:**
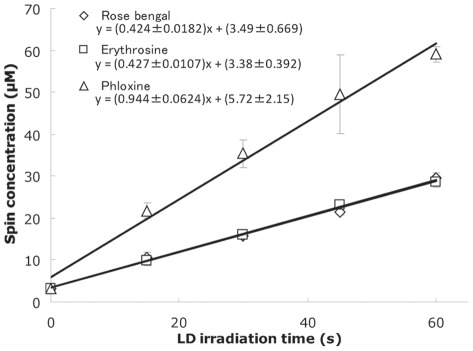
The yields of the nitroxide radical from the laser-irradiated photosensitizers. The mixture containing 450 mM TPC and 10 µM photosensitizer was irradiated by the laser light with a wavelength of 532 nm for 15, 30, 45 and 60 s. Each value represents the mean of triplicate determinations with standard deviation. The slope and intercept of each regression line are expressed as the mean with standard error.

Representative ESR spectra of the laser-irradiated mixtures containing different concentration of rose bengal and 2,2,5,5-tetramethyl-3-pyrroline-3-carboxamide (TPC) are shown in Fig. 1. When a mixture containing 450 mM TPC and 1 or 10 µM rose bengal was irradiated by the laser light with a wavelength of 532 nm for 60 s, a typical signal of the nitroxide radical generated during the oxidation of TPC by singlet oxygen was observed dependently on the concentration of rose bengal as described in our previous study [23]. When the singlet oxygen producing abilities of the three photosensitizers, rose bengal, erythrosine, and phloxine at a fixed concentration of 10 μM were examined, the amounts of nitroxide radical increased linearly with the laser irradiation time at least up to 60 s (Fig. 2). The generation rates of nitroxide radical which corresponded to the slope values of the regression lines for the two variables, time and spin concentration, were 0.42±0.02 (mean ± standard error) µMs^−1^, 0.43±0.01 µMs^−1^, and 0.94±0.06 µMs^−1^ for rose bengal, erythrosine, and phloxine, respectively, indicating that the yield of the radical was in the order phloxine > erythrosine ≒ rose bengal.

### Bactericidal activity of laser-irradiated photosensitizers

**Figure 3:**
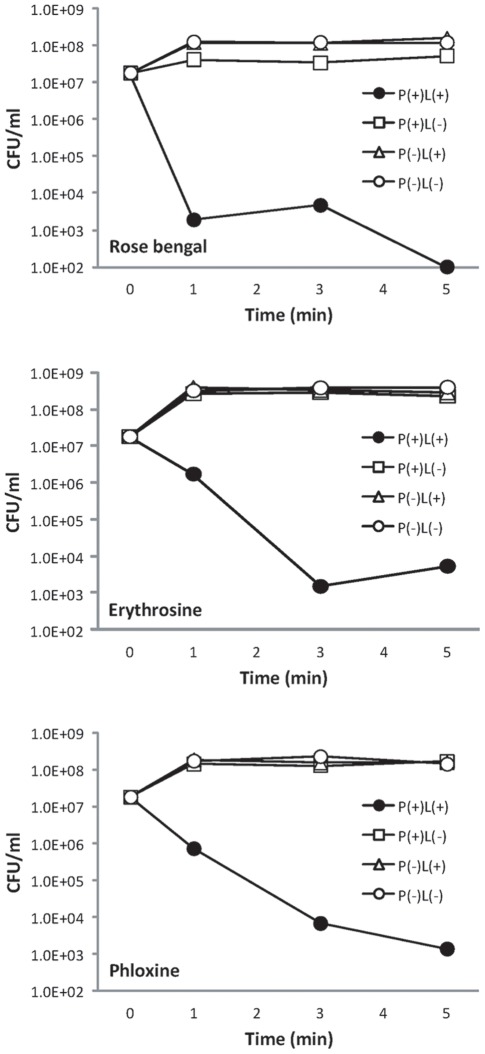
Number of viable *S. mutans* in the suspension after each treatment. P(+)L(+), P(+)L(−), P(−)L(+), and P(−)L(−) stand for laser irradiation of each photosensitizer, photosensitizer alone, laser irradiation alone, and no treatment, respectively. Under the conditions of P(+)L(+) and P(+)L(−), the suspension contained 10 μM rose bengal, phloxine, or erythrosine. Each value represents the mean of nonuplicate determinations.

All of the photosensitizers excited by laser irradiation killed effectively the bacterial strain used in the present study in an irradiation-time dependent manner, and the numbers of bacteria were reduced by 3 or more logarithmic order within 3 min (Fig. 3). Of the photosensitizers, rose bengal exerted the highest activity against the bacterial strain with 3 logarithmic reduction within a short time as 1 min, and phloxine and erythrosine showed almost the same activity with approximately 1 logarithmic reduction within 1 min. Under the other three conditions expressed as P(+)L(−): photosensitizer alone, P(−)L(+): laser irradiation alone, and P(−)L(−): no treatment, almost no bactericidal effects were observed.

**Figure 4:**
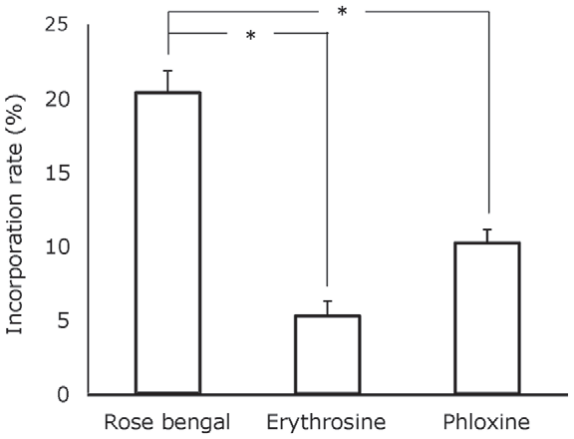
Incorporation rates of photosensitizers into the bacterial cells. The bacterial suspension containing 10 μM rose bengal, phloxine, or erythrosine was incubated at room temperature for 3 min. Each value represents the mean of triplicate determinations with standard deviation. Statistically significant differences between the two groups are shown as * (p<0.05).

To further clarify the relation between the singlet oxygen producing ability and the bactericidal activity, incorporation efficiency of these photosensitizers into bacterial cells was also examined (Fig. 4). Of the three photosensitizers examined in the present study, the highest incorporation rate was obtained in rose bengal, followed by phloxine and erythrosine. That is, 20% of the rose bengal added to the bacterial suspension was incorporated into the bacterial cells, and 10% and 5% for the phloxine and the erythrosine, respectively. Statistically significant differences between the two groups were found in all combinations.

**Figure 5:**
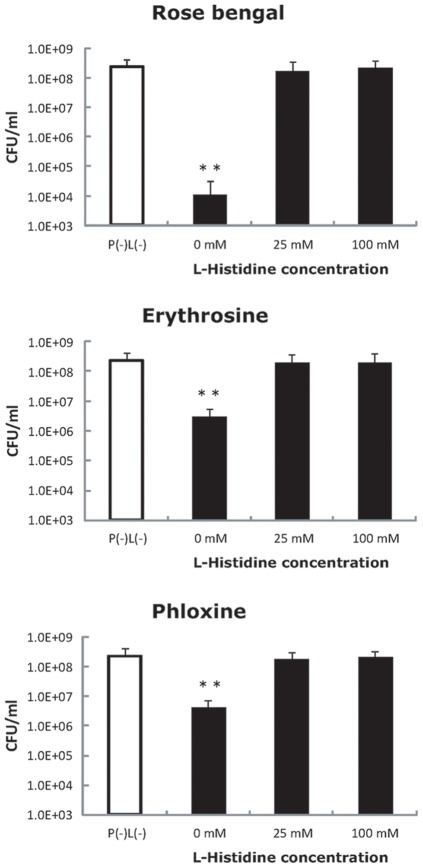
Influence of L-histidine, a singlet oxygen quencher, on the bactericidal effects of laser-irradiated photosensitizers. L-Histidine (25 and 100 mM) was added to the bacterial suspension containing 10 μM rose bengal, phloxine, or erythrosine, followed by laser-irradiation for 60 s. Each vealue represents the mean of nonuplicate determinations with standard deviation. Statistically significant differences from the corresponding P (−)L(−) group are shown as ** (p<0.01).

To confirm if the bactericidal effect obtained under the condition of P(+)L(+) was attributable to singlet oxygen generated by the laser-irradiated photosensitizers, effect of L-histidine, a singlet oxygen quencher, was examined (Fig. 5). Addition of not only 100 mM but 25 mM L-histidine completely abolished the bactericidal effect of laser-irradiated photosensitizers.

## Discussion

Nitroxide radical generation through oxidation of TPC is illustrated in Fig. 1. When singlet oxygen is photogenerated, the proton is subtracted from the amine moiety of TPC concomitant with oxygen accretion reaction yielding the corresponding nitroxide radical as detected by the ESR analysis (Fig. 1). Since the amount of nitroxide radical increased with laser-irradiation time (Fig. 2), it was confirmed that singlet oxygen was generated time-dependently on the laser-irradiation of the photosensitizers, rose bengal, phloxine, and erythrosine. Regarding the singlet oxygen generation efficiency, it was proved that of the three photosensitizes phloxine has the highest ability to generate singlet oxygen because the slope value of the regression line as shown in Fig. 2 was the highest followed by those of rose bengal and erythrosine. Therefore, if the bactericidal effect of laser-irradiated photosensitizers had been solely dependent on the amount of photogenerated singlet oxygen, phloxine would have shown the highest bactericidal activity. However, since rose bengal but not phloxine showed the highest bactericidal activity (Fig. 3), factors other than singlet oxygen also seemed to be involved in the bactericidal activity. The most likely factor is incorporation efficiency of the photosensitizers into the bacterial cells. Indeed, rose bengal was most efficiently incorporated into the bacterial cells as compared to the other two photosensitizers (Fig. 4), likely leading to the highest bactericidal activity. Meanwhile, the bactericidal activity of laser-irradiated phloxine was almost the same as that of erythrosine, even though both the singlet oxygen producing ability and the incorporation efficiency of phloxine were superior to those of erythrosine. One of the possibilities is that erythrosine itself would have cytotoxic effect on the bacterial cells in some degree. However, no bactericidal effect of erythrosine was observed under the condition of P(+)L(−), suggesting that, even if erythrosine is cytotoxic, cytotoxic property of erythrosine is negligible. The other possibility is localization of the incorporated photosensitizers in the bacterial cells. That is, if a photosensitizer is mainly localized only around the cell wall, photogenerated singlet oxygen fails to damage enzymes, nucleic acids, and subcellular organelles in the cytoplasm. The aspect of the localization at cellular level is the next issue to be studied in the future. Finally, to prove that the bactericidal effect of the excited photosensitizers would be attributable to photogenerated singlet oxygen, effect of L-histidine, a singlet oxygen quencher, was examined. Since the addition of 25 mg/ml of L-histidine cancelled the bactericidal activity of any of the three excited photosensitizers (Fig. 5), it is proved that singlet oxygen is responsible for the bactericidal action elicited by the laser-irradiated photosensitizers. From these, it is strongly suggested that rose bengal is a suitable photosensitizer for the plaque disclosing agents as compared to the other two photosensitizers, phloxine and erythrosine, when used for PDT.

## Materials and Methods

### Reagents

Reagents were purchased from the following sources: TPC, and 4-hydroxy-2,2,6,6-tetramethylpiperidine 1-oxyl (TEMPOL) from Sigma Aldrich (St. Louis, MO, USA); rose bengal, erythrosine, and phloxine from Wako Pure Chemical Industries (Osaka, Japan). All other reagents used were of analytical grade.

### An experimental laser device for excitation of photosensitizers

To determine a wavelength of laser light for excitation of rose bengal, erythrosine, and phloxine, maximal absorption wavelengths of the three photosensitizers were spectrophotometrically determined. That is, 550 nm, 530 nm, and 540 nm for rose bengal, erythrosine, and phloxine, respectively, were obtained, indicating that the wavelengths of 530 to 550 nm are suitable to properly excite all of the three photosensitizers. An experimental laser device equipped with the second harmonic of Nd-YAG laser (wavelength: 532 nm) and a laser power meter was made (PAX Co. Ltd., Sendai, Japan) as described in our previous study [23]. An output power of the laser was set at 20 mW in the present study. When a semi-micro cuvette containing 200 µl of sample is set in the experimental device, the area of the sample irradiated by the laser is almost 5×5 mm resulting in the energy dose of 80 mW/cm^2^. The light path of the cuvette was 10 mm.

### Determination of photogenerated singlet oxygen by ESR analysis of nitroxide radical

A sterically hindered amine, TPC, was used without further purification. The TPC was dissolved in ultrapure water to make a 500 mM solution. Each photosensitizer (rose bengal, erythrosine, and phloxine) was dissolved in ultrapure water to make 100 µM solutions. Then, 180 µl of TPC solution and 20 µl of each photosensitizer solution were mixed in a disposable plastic semi-micro cuvette to make final concentrations of 450 mM for TPC, and 10 µM for the photosensitizer. In an experiment conducted to check the ESR spectra, final concentrations of 1 and 10 µM rose bengal were used. Immediately after mixing, the cuvette was set in the experimental laser device. The sample in the cuvette was irradiated by the laser light for 60 sec. After the laser irradiation, the sample was transferred to a quartz cell and ESR spectrum was recorded on an X-band ESR spectrometer (JES-FA-100, JEOL, Tokyo, Japan). The measurement conditions for ESR were as follows: field sweep, 330.50–340.50 mT; field modulation frequency, 100 kHz; field modulation width, 0.05 mT; amplitude, 80; sweep time, 2 min; time constant, 0.03 sec; microwave frequency, 9.420 GHz; microwave power, 4 mW. To calculate the spin concentration of the nitroxide radical, which was generated through oxidation of TPC by singlet oxygen, 20 µM TEMPOL was used as a standard sample and the ESR spectrum of manganese (Mn^2+^) which was equipped in the ESR cavity was used as an internal standard. The spin concentration was determined using Digital Data Processing (JEOL, Tokyo, Japan). All tests were performed in triplicate.

### Bactericidal test

Stock culture strains of *Streptococcus mutans* JCM 5705 was purchased from Japan Collection of Microorganisms, RIKEN BioResource Center (Wako, Japan). The bacterial strain was cultured on Brain Heart Infusion (BHI) agar (Becton Dickinson Labware, Franklin lakes, NJ, USA) anaerobically using Anaero Pack (Mitsubishi Gas Chemical Company, Tokyo, Japan) at 37°C. The bacterial suspension was prepared in sterile physiological saline from cultures grown on BHI agar at 37°C for 2 days. The suspension was adjusted to approximately 2×10^7^ colony forming units (CFU)/ml. The bactericidal potency of the laser-irradiated photosensitizer was examined. An aliquot (180 µl) of the bacterial suspension and 20 µl of each photosensitizer solution prepared as described above were mixed in a disposable plastic semi-micro cuvette to make final concentration of 10 µM for the photosensitizer. Immediately after mixing, the cuvette was set in the experimental laser device. The sample in the cuvette was irradiated by the laser light for 1, 3, and 5 min. After the irradiation, a ten-fold serial dilution of the mixture was prepared using sterile physiological saline and 10 µl of the dilution was seeded onto BHI agar to determine the number of viable bacterial cells in the suspension. The agar medium was cultured for 48 h under the conditions as described above. The number of CFU/ml was then determined. The bactericidal effect of laser irradiation of each photosensitizer (expressed as P(+)L(+)) was compared to the effect of; (1) photosensitizer alone, P(+)L(−), (2) laser irradiation alone, P(−)L(+), and (3) no treatment, P(−)L(−). For the condition of L (−), the samples were kept without the laser irradiation in the clean bench to avoid contamination. For the condition of P(−), sterile physiological saline was added into the reaction system instead of the photosensitizer solution. All tests were repeatedly conducted nine times.

### Effect of singlet oxygen quencher on bactericidal activity

To further examine if bactericidal effects of laser-irradiated phostosensitozers were attributable to the generated singlet oxygen, L-histidine, a singlet oxygen quencher [26], [27], was added to the reaction mixture of bacterial suspension and photosensitizer. More in detail, 100 μl of the bacterial suspension (*S. mutans* JCM 5705), 20 μl of photosensitizer, and 80 μl of L-histidine was mixed in a disposable plastic semi-micro cuvette to make final concentrations of 2.5×10^8^ CFU/ml for the bacteria, 10 μM for the photosensitizer, and 25 or 100 mM for L-histidine. Immediately after mixing, the cuvette was set in the experimental laser device. The sample in the cuvette was irradiated by the laser light for 60 sec. After the irradiation, the number of CFU/ml in the suspension was determined as described above. All tests were repeatedly conducted nine times. Statistical analysis for the significant differences from the mean values under the condition of P(−)L(−) was done by Dunnett's multiple comparison test following logarithmic conversion. P values of <0.05 were considered significant.

### Incorporation efficiency of photosensitizer into bacterial cells

An aliquot (1800 μl) of *S. mutans* suspension (approximately 10^8^ CFU/ml) and 200 μl of 100 μM photosensitizer were mixed and incubated for 3 min at room temperature under a light shielding condition. Then the bacteria were harvested by centrifugation at 4000 x g for 5 min. Following decantation of the supernatant, the pellet was washed twice by physiological saline. For the extraction of the photosensitizer, 200 μl of 99.5% ethanol was added to the resultant bacterial pellet, followed by vigorous agitation. Following centrifugation, absorbance of the supernatant was determined at the maximal absorbance wavelength of each photosensitizer. Incorporation rates of the photosensitizers were calculated by the following equation. That is (incorporated amount of photosensitizer/added photosensitizer) ×100. All tests were performed in triplicate. Statistical analysis for the incorporation rates was done by Tukey-Kramer's multiple comparison test. P values of <0.05 were considered significant.
